# Tetris and Word games lead to fewer intrusive memories when applied several days after analogue trauma

**DOI:** 10.1080/20008198.2017.1386959

**Published:** 2017-10-31

**Authors:** Muriel A. Hagenaars, Emily A. Holmes, Fayette Klaassen, Bernet Elzinga

**Affiliations:** ^a^ Leiden Institute for Brain and Cognition (LIBC), Leiden, The Netherlands; ^b^ Department of Clinical Psychology, Utrecht University, Utrecht, The Netherlands; ^c^ Department of Clinical Neuroscience, Karolinska Institutet, Stockholm, Sweden; ^d^ Department of Methodology and Statistics, Utrecht University, Utrecht, The Netherlands; ^e^ Department of Clinical Psychology, Leiden University, Leiden, The Netherlands

**Keywords:** Trauma film, posttraumatic stress disorder, PTSD, reconsolidation, intrusions, intrusive memory, involuntary memory, mental imagery, working memory, • A trauma film paradigm was used to examine effects of a supposedly visuospatial (Tetris) and a more verbal task (Word games) versus no task (reactivation-only) on intrusion frequency in a reconsolidation time frame., • Reactivation+Tetris and reactivation+Word games lead to relatively fewer intrusions than reactivation-only, even when applied four days after analogue trauma., • Two hypotheses were supported: (a) There is an intervention effect with both task conditions being equally effective (reactivation+Tetris = reactivation+Word games < reactivation-only), and (b) There is a modality effect with Word games being the most effective task (reactivation+Word games < reactivation+Tetris < reactivation-only)., • Participants rated Word games as more difficult than Tetris, but there was no difference in intrusion frequencies during the performance of both tasks. • Tetris and Word games lead to fewer intrusive memories when applied several days after analogue trauma.

## Abstract

**Background**: Intrusive trauma memories are a key symptom of posttraumatic stress disorder (PTSD), so disrupting their recurrence is highly important. Intrusion development was hindered by visuospatial interventions administered up to 24 hours after analogue trauma. It is unknown whether interventions can be applied later, and whether modality or working-memory load are crucial factors.

**Objectives**: This study tested: (1) whether a visuospatial task would lead to fewer intrusions compared to a reactivation-only group when applied after memory reactivation four days after analogue trauma exposure (extended replication), (2) whether both tasks (i.e. one aimed to be visuospatial, one more verbal) would lead to fewer intrusions than the reactivation-only group (intervention effect), and (3) whether supposed task modality (visuospatial or verbal) is a critical component (modality effect).

**Method**: Fifty-four participants were randomly assigned to reactivation+Tetris (visuospatial), reactivation+Word games (verbal), or reactivation-only (no task). They watched an aversive film (day 0) and recorded intrusive memories of the film in diary A. On day 4, memory was reactivated, after which participants played Tetris, Word games, or had no task for 10 minutes. They then kept a second diary (B). Informative hypotheses were evaluated using Bayes factors.

**Results**: Reactivation+Tetris and reactivation+Word games resulted in relatively fewer intrusions from the last day of diary A to the first day of diary B than reactivation-only (objective 1 and 2). Thus, both tasks were effective even when applied days after analogue trauma. Reactivation-only was not effective. Reactivation+Word games appeared to result in fewer intrusions than reactivation+Tetris (objective 3; modality effect), but this evidence was weak. Explorative analyses showed that Word games were more difficult than Tetris.

**Conclusions**: Applying a task four days after the trauma film (during memory reconsolidation) was effective. The modality versus working-memory load issue is inconclusive.

## Abstract (es)

**Planteamiento:** Los recuerdos intrusivos de trauma son un síntoma clave del trastorno de estrés postraumático (TEPT), por lo que es muy importante interrumpir su recurrencia. Se obstaculizó el desarrollo de intrusiones mediante intervenciones visuoespaciales administradas hasta 24 horas después de un trauma analógico. Se desconoce si las intervenciones pueden ser aplicadas más adelante, y si la modalidad o la carga de la memoria de trabajo son factores cruciales.

**Objetivos:** El estudio comprobaba: (1) si una tarea visuoespacial conduciría a menos intrusiones en comparación con un grupo de solo reactivación cuando se aplicaba después de la reactivación del recuerdo, 4 días después de la exposición al trauma analógico (replicación extendida); (2) si ambas tareas (una pretendía ser más visuoespacial y otra más verbal) darían lugar a menos intrusiones que el grupo de solo reactivación (efecto de la intervención), y 3) si la supuesta modalidad de la tarea (visuoespacial o verbal) es un componente fundamental (efecto de la modalidad).

**Método:** Se asignaron aleatoriamente 54 participantes a reactivación + Tetris (visuoespacial), reactivación + juegos de palabras (verbal), o solo reactivación (sin tarea). Vieron una película aversiva (día 0) y registraron recuerdos intrusivos de la película en el diario A. El cuarto día, se reactivó el recuerdo, después de lo cual los participantes jugaron al Tetris, a juegos de palabras, o no hicieron ninguna tarea durante 10 minutos. Luego escribieron un segundo diario (B). Se evaluaron las hipótesis informativas con factores de Bayes.

**Resultados:** Reactivación + Tetris y reactivación + juegos de palabras dieron como resultado relativamente menos intrusiones que sólo la reactivación (objetivo 1 y 2), desde el último día del diario A hasta el primer día del diario B. Por lo tanto, ambas tareas fueron eficaces incluso cuando se aplicaron días después del trauma analógico. La reactivación por separado no fue efectiva. Reactivación + juegos de palabras pareció resultar en menos intrusiones que reactivación + Tetris (objetivo 3, efecto de la modalidad), pero esta evidencia era débil. Los análisis exploratorios mostraron que los juegos de palabras eran más difíciles que el Tetris.

**Conclusiones:** La aplicación de una tarea 4 días después de la película traumática (durante la reconsolidación de la memoria) fue efectiva. La modalidad versus el problema de la carga de memoria de trabajo no es concluyente.

## Abstract (zh)

**背景：**闯入创伤记忆是创伤后应激障碍（PTSD）的关键症状，所以阻断其反复出现十分重要。闯入症状的发展可以被模拟创伤之后24小时以内的视觉空间干预阻断。但是还不清楚干预是否可以在更晚的时间施行，以及通道或者工作记忆负荷是否是关键因素。

**目标：**本研究考察1）在模拟创伤暴露4天后，视觉空间任务是否可以比仅进行再激活的小组更少闯入（扩展重复）；2）是否两种任务（比如一种集中在视觉空间，一种更偏向言语）是否会比仅进行再激活引起更少的闯入（干预效果）；3）是否预期的任务通道（视觉空间或者言语）是关键成分（通道效应）。

**方法：**54名被试随机分配到‘再激活+俄罗斯方块’（空间视觉）组，‘再激活+文字游戏’（言语）组或者‘只有再激活’（无任务）组。被试观看一段厌恶刺激影片（第0天）并在日记A中记录关于影片的闯入记忆。在第4天重复激活记忆，此后让被试玩十分钟俄罗斯方块、文字游戏或者没有任务，之后被试记录日记B。使用贝叶斯因子检验信息化假设。

**结果：**和‘只有再激活’组相比，‘再激活+俄罗斯方块’组和‘再激活+文字游戏’组在B日记的第一天和A日记的最后一天相比报告相对更少的闯入（目标1和2）。因此在模拟创伤的几天后进行的两种任务都是有效的。‘只有再激活’组没有效果。‘再激活+文字游戏’比‘再激活+俄罗斯方块’的闯入更少（目标3，通道效应），但证据较弱。探索性分析显示文字游戏比俄罗斯方块更难。

Many people experience trauma in the course of their lives and quite some of those – about 7.8% (Perkonigg, Kessler, Storz, & Wittchen, 2000) – develop posttraumatic stress disorder (PTSD). PTSD is a disorder characterized by intrusive memories, avoidance, and negative alterations in cognitions, mood, arousal, and reactivity (American Psychiatric Association, 2013). PTSD is one of the few psychiatric disorders with a clear onset event (the trauma), and thus prevention strategies would be relatively applicable. However, to date effective preventive interventions are lacking, although some studies have found trauma focused Cognitive Behavioural Therapy (CBT) to be effective (Roberts, Kitchiner, Kenardy, & Bisson, 2010; Rothbaum et al., 2012; Sijbrandij et al., 2007).

Adequate prevention/early intervention strategies would preferably tap into the key characteristic of PTSD: vivid, distressing intrusive memories of the trauma (Brewin, 2015). Cognitive models for PTSD propose that intrusive memories are a core symptom (Brewin, 2001; Ehlers & Clark, 2000), and there is indeed some empirical evidence that early intrusive memories (in the first days) contribute to PTSD development (Creamer, O’Donnell, & Pattison, 2004; O’Donnell, Elliott, Lau, & Creamer, 2007).

Laboratory studies can be helpful here, as these allow investigation of the effects of different interventions in a controlled way as well as exploring underlying mechanisms of change. The trauma-film paradigm is most frequently used for this purpose. This paradigm typically includes analogue trauma (i.e. an aversive film) and intrusive memories as the main outcome variable and is therefore a useful experimental psychopathology model for PTSD (Holmes & Bourne, 2008; James et al., 2016). Several laboratory-based interventions indeed seem promising in preventing or altering memories for analogue trauma. For example, playing the computer game Tetris immediately after analogue trauma reduced the frequency of intrusive memories of the aversive film immediately (Deeprose, Zhang, Dejong, Dalgliesh & Holmes, 2012), after 30 minutes (Holmes, James, Coode-Bate, & Deeprose, 2009; Holmes, James, Kilford, & Deeprose, 2010, experiment 1), and after four hours (Holmes et al., 2010, experiment 2), as did imagery rescripting after 30 minutes (Hagenaars, 2012; Hagenaars & Arntz, 2012).

Laboratory interventions have typically been applied during or soon after the analogue trauma, thereby disrupting memory encoding or consolidation, thus hypothesized to disrupt initial acquisition of a memory trace and/or its stabilization (or consolidation) into long-term memory (Dudai, 2004). Memories are malleable during the consolidation phase, which is thought to end after approximately six hours (Nader, 2003; Walker, Brakefield, Hobson, & Stickgold, 2003). The memory is then proposed to be stable, i.e. no longer actively formed or changed except if the traumatic event is retrieved, for instance during recollection triggered by event-related cues (Nader & Hardt, 2009), a process hitherto referred to as ‘memory reactivation’.

During this retrieval phase, trauma memories are thought to be retrieved in working memory and, as a consequence, become partly malleable again, providing the opportunity to modify them (Nader & Hardt, 2009; Van den Hout & Engelhard, 2012). After retrieval, memories are stored again: *re*consolidation. Once a memory is reconsolidated, the new trace becomes stable again (Dudai, 2004). Most experiments have targeted memories of analogue trauma in the consolidation phase (for reviews see Holmes & Bourne, 2008; James et al., 2016). A recent study stretched the post-trauma intervention to 24 hours post-trauma (James et al., 2015). The authors found that it was still effective, but only if memory was activated 10 minutes *before* the intervention, consistent with the hypothesis that reconsolidation processes are at stake here (Debiec et al., 2002; Dudai, 2004). This is in line with fear conditioning studies, where propranolol proved to reduce fear responses when administered before memory reactivation (i.e. the reconsolidation phase, 24 hours after acquisition; Kindt, Soeter, & Vervliet, 2009).

In daily life, interventions may not be administered within a few hours – or even 24 hours – after actual trauma. However, they may more readily be applied later, when the trauma memory is already consolidated. Therefore, it is important to establish whether the time between trauma and intervention can be increased. A first step would be to test whether analogue trauma memories can indeed be altered several days after an analogue trauma, i.e. during memory reconsolidation. We therefore stretched the time between encoding of the analogue trauma (an aversive film) and the administration of a frequently used intervention that was effective in influencing the process of reconsolidation (Tetris) to four days.

Dual processing theories that distinguish perceptual and conceptual memory (Brewin, Dalgleish, & Joseph, 1996; Ehlers & Clark, 2000; Holmes & Bourne, 2008) posit that dominant perceptual information processing leads to the development of intrusions. To test this, laboratory interventions have been designed to tap into one or other of these memory systems. Visuospatial interventions use resources needed for perceptual processing hence should lead to a shift towards conceptual processing and decreased intrusions, and vice versa for verbal interventions. Note such verbal interventions are typically not related to the analogue trauma content, because then other mechanisms would be at stake (e.g. devaluation or changing appraisals; Ehlers & Clark, 2000). Interventions that tax visuospatial processing during or after analogue trauma have indeed been found to be consistently successful in reducing subsequent intrusion frequencies over the subsequent week (e.g. Tetris or complex pattern finger tapping; Holmes, Brewin & Hennessy, 2004; James et al., 2015). The findings on verbal tasks are less consistent. Verbal interventions increased intrusion frequencies in some studies (counting backwards in threes/sevens: Bourne, Frasquilho, Roth, & Holmes, 2010, exp 2; Holmes et al., 2004; Pub quiz: Holmes et al., 2010, exp 1), but not in others (counting backwards in threes/sevens: Deeprose et al., 2012; Krans, Näring, & Becker, 2009; Pub quiz: Holmes et al., 2010, exp 2; verbalize what goes through your mind while watching the film: Holmes et al., 2004; for a review, see Brewin, 2014; James et al., 2016), and were also found to decrease intrusion frequency (verbalize what goes through your mind while watching the film: Krans et al., 2009). This may partly be explained by the large variety of verbal tasks that have been used. One could wonder whether these verbal tasks (e.g. counting backwards) sufficiently tap into conceptual memory and involve contextual processing (Holmes & Bourne, 2008). To examine the effect of a clearly verbal task on reconsolidation of an analogue trauma memory, a word game was developed that draws on verbal resources by demanding an active mental search for *words* instead of numbers.

A task may retroactively interfere with memory when executed within a certain time frame, especially with young memory traces (Wixted, 2004). Concurrent tasks seem to interfere with each other, influencing memory (re)consolidation. Interpretations of the working memory theory in this context posit that any concurrent task that taxes the central-executive component of working memory would interfere with reconsolidation, leading to reduced emotionality and vividness of the original memory (Baddeley, 1998; Van den Hout & Engelhard, 2012). Working memory is considered to be a four-component system including visuo-spatial sketch pad, phonological loop, episodic buffer and central executive (Baddeley, 2012). The central executive is the centre of the system that carries out higher order cognitive processing (e.g. focusing attentional, dividing attention, planning). Thus, according to such an interpretation of the working memory theory (Van den Hout & Engelhard, 2012), working memory load rather than modality is the crucial component (Gunter & Bodner, 2008). Moreover, working memory theory also implies a dose–response relationship with stronger effects for more demanding tasks, which has indeed been found in empirical studies (Engelhard, Van den Hout, & Smeets, 2011; Van den Hout & Engelhard, 2012). The working memory theory has been tested frequently using different dual tasks such as eye movements, auditory shadowing, drawing and calculating out loud and mental arithmetics, which indeed reduced memory emotionality and vividness (e.g. Engelhard et al., 2011; Gunter & Bodner, 2008; Kemps & Tiggemann, 2007). Note however that these experiments typically use autobiographical memories (assessing mostly immediate effects on voluntary memory characteristics) and not a trauma film paradigm in which involuntary memories (intrusions) are experimentally generated. In addition, they typically use tasks as a dual task (i.e. tasks are executed simultaneously while recalling the autobiographical memory and thus proposedly competing for working memory resources) whereas tasks in a trauma film paradigm are typically performed after memory reactivation.

The first aim of this study was to test whether memory reactivation followed by Tetris gameplay (reactivation+Tetris) would lead to fewer intrusive memories compared to a reactivation-only group, when applied as long as four days after analogue trauma (film). To address this aim, Tetris was tested separately against the reactivation-only control condition, in order to examine whether previous findings could be replicated when applying this previously effective intervention longer after analogue trauma. Second, we aimed to test whether both tasks (i.e. one visuospatial, one verbal; reactivation+Tetris and reactivation+Word games) would result in relatively fewer intrusive memories (intervention effect) compared to a no-task control group (reactivation-only). The third aim was to examine whether task modality is a crucial component (i.e. difference between reactivation+Tetris and reactivation+Word games; modality effect).

We expected small effects, given that the trauma film is analogue of real trauma and participants are healthy, and intrusions usually diminish quickly over time after watching an aversive film. However, given the consistent findings with Tetris, we did expect a small effect of reactivation+Tetris after four days (aim 1) compared to reactivation-only controls. With regard to the second and third aim: according to original dual processing theories, an effect of modality would be expected (i.e. reactivation+Tetris more effective than reactivation+Word games), whereas working memory theories would predict that both interventions are effective, provided they have the same working memory load. As working memory theory predicts more impact for tasks with higher working memory load, we additionally explored the role of task difficulty as indicated by self-reported ratings and intrusions during the task (Holmes et al., 2009). Ratings of task pleasantness were also obtained because pleasant tasks may affect trauma memory by means of devaluating the trauma (Pile, Barnhofer, & Wild, 2015; Zbozinek, Holmes, & Craske, 2015).

We used Bayesian statistics and not the usual frequentist approach for several reasons. First, we aimed to test several competing informative hypotheses against each other (specific hypotheses are listed in the Method section). Second, we aimed to find evidence for or against the null-hypothesis, which can be achieved using a Bayesian analysis. Finally, a Bayesian approach allows for building cumulative evidence across studies.

## 1. Method

### 1.1. Participants

Sixty-one Dutch students participated in the study with an age range of 18–41 years (40 females; mean age 22.2 years; SD 3.76). Exclusion criteria were: presence of PTSD, frequent Tetris player, having been involved in a motor vehicle accident recently (the trauma film contained road traffic accidents and we did not want to reactivate participant’s autobiographical memories). Two participants ended the experiment during the first appointment (they entered the laboratory feeling ill). Four participants dropped out after the first appointment (one forgot the appointment and the others did not want to proceed). One participant did not show up for the third appointment. These seven participants were excluded from analyses leaving 18 participants in each group (*N* = 54). Group allocation was random. All participants provided their written informed consent, in which the aversive film was mentioned. Participants received either seven study credits or 14 Euros for their participation.

### 1.2. Material

#### 1.2.1. Trauma film

A 11.5-minute video comprising four scenes with real-life footage (compiled by Steil, 1996) was shown as analogue trauma. The original film consisted of five scenes of live footage from the aftermath of road traffic accidents, including emergency service personnel working to extract trapped victims, injured victims moaning, and dead bodies being moved. Before each scene, a brief commentary provided context to each accident and the people involved. Following Hagenaars, Van Minnen, Holmes, Brewin, and Hoogduin (2008), the least distressing scene was removed from the original five scenes. The film was shown on a 15-inch computer screen with 45 cm distance between the screen and the participant.

#### 1.2.2. General stress symptoms

The Symptom Check List-90 (SCL-90; Derogatis, 1992) was used to measure various psychopathological symptoms that participants experienced in the previous week. The 90 self-report items of the SCL-90 are scored on a 5-point Likert scale. The SCL-90 has nine subscales assessing Somatization, Obsessive-Compulsive, Interpersonal Sensitivity, Depression, Anxiety, Anger-Hostility, Phobic Anxiety, Paranoid Ideation, and Psychoticism.

#### 1.2.3. Distress

Participants rated the level distress on a scale from 0 (not at all) tot 10 (extremely) before and directly after the film.

#### 1.2.4. Attention to the film

Participants rated the extent to which they paid attention to the film on a scale from 0 (‘not at all’) to 10 (‘extremely’).

#### 1.2.5. Attention to the memory reactivation stills

Participants also rated the extent to which they paid attention to the still images that were shown in the memory reactivation task from 0 (‘not at all’) to 10 (‘extremely’).

#### 1.2.6. Intrusive memories

Participants recorded intrusive memories of the film in a tabular diary daily. Intrusions were defined as spontaneously occurring image-based intrusions of the trauma film (Holmes et al. 2004; Hagenaars, Brewin, Van Minnen, Holmes, & Hoogduin, 2010; Hagenaars et al., 2008; Holmes et al., 2009). For every intrusion, participants had to record intrusion-type (image-based or thought-based), the content of the intrusions, the situation they were in at the time of the intrusion, the emotions associated with the intrusion (happy, sad, angry, afraid, disgust, neutral), how much stress it evoked (on a scale from 0 to 100), sense of control over the intrusion (0–100), its vividness (0–100), and how spontaneous it came up (0–100). If participants had experienced no intrusions during a certain period, they recorded 0. Clear standardized written instructions were given about how to keep the diary. The participants were asked to carry the diary with them at all times and to record each intrusion as soon as possible after occurrence. They were also asked to dedicate a moment each day to check whether they had completed their diary and to indicate whether they had any intrusions.

There were two diaries: diary A started immediately after experimental session 1 (day 0) and ended at experimental session 2 (day 4). Diary B started immediately after experimental session 2 (day 4) and ended at experimental session 3 (day 7). Intrusions were checked at experimental session 2 (diary A) and session 3 (diary B).

As we were interested in the change in intrusive memories from before to after the intervention, we used the change in the number of intrusions from diary A (last day) to diary B (first day) was selected as main outcome variable. This change score was calculated by subtracting the number of intrusions for the last day in diary A from the first day in diary B. This way, we controlled for small and insignificant differences in diary A. Positive scores on this change-variable reflect an increase in the number of intrusions after reactivation/intervention. Intrusion frequencies usually rapidly decrease in the days following a trauma film (diary A; see also James et al., 2015 for time series analysis in a day-by-day decline in intrusions over one week). We expected low intrusion frequencies in diary B, and minimal intrusions after the first day of diary B. We therefore decided posthoc to use the first day of diary B rather than the total frequency, because the latter would be too sensitive to accidental and not task-related fluctuations in just individual participants. This would give the best indication of the task effects given the current design.

#### 1.2.7. Diary compliance

A compliance question was asked after each diary was handed in: ‘I was often not capable of (or I forgot) recording my intrusions in my diary’ (0 [not at all true] to 10 [very true]). Thus, a higher score means less compliance. Compliance to diary A and diary B give an indication of the reliability of the self-reported intrusions in the diaries.

#### 1.2.8. Memory reactivation

In experimental session 2, participants were shown four still film images for seven seconds each, containing characteristic shots of each scene of the trauma film. An example is a woman sitting stuck in a van just before she was carried out by paramedics. These stills were meant to activate the memory of the film, but there was no explicit instruction of reactivating the memory.

#### 1.2.9. Intrusive memories during performance of the task

During the 10-minute period after memory reactivation (thus during Tetris, Word games or sitting quietly) participants in all conditions recorded any occurrence of an intrusive memory of the film on a sheet of paper lying next to the computer (frequency only). The total intrusion frequency was used as an index of task difficulty, given that a more difficult task (i.e. higher executive demand on working memory resources; Baddeley, 2000) would leave less room for intrusive memories.

#### 1.2.10. Task difficulty and pleasantness

Difficulty and pleasantness of the task (Tetris and Word games) were rated after the task on a scale from 0 (‘not at all difficult’ and ‘not at all pleasant’) to 10 (‘extremely difficult’ and ‘extremely pleasant’).

### 1.3. Experimental tasks

Right before the second experimental session participants were randomly assigned to one of the three conditions, comprising memory reactivation followed by a task: reactivation+Visuospatial Task (Tetris), reactivation+Verbal Task (Word games), and reactivation-only. The experiment was exactly the same for all conditions except for the tasks after memory reactivation.

#### 1.3.1. Reactivation+Tetris

After the memory reactivation, participants played the game Tetris on a computer for 10 minutes. They used the cursor keys to move and rotate falling blocks to complete the largest number of complete rows across the screen (Crystal Office Systems, 2015). The game has 10 speed levels; during level 1 the blocks fall slowly and during level 10 they fall extremely fast. All participants started at level 3. If they failed the game before 10 minutes had passed, the experimenter reset the game at level 2. In that case, participants played Tetris at level 2 for the time that was left.

#### 1.3.2. Reactivation+Word games

After the memory reactivation participants played two word games. In the first game they had to form as many Dutch words as possible with 10 randomly chosen letters (e.g. D, E, J, K, L, N, O, O, U, S). In the second game they had to make as many words as possible starting with specific letters (e.g. SL…). Both games started with an example and then lasted five minutes. Each minute a different level started automatically. Participants used paper and pencil to write down their answers.

#### 1.3.3. Reactivation-only control condition

Participants sat quietly in their seats for 10 minutes. They were asked to stay seated, to leave their mobile phones off and not to speak during this period. The experimenter monitored the participants from behind a one-way screen.

### 1.4. Procedure

#### 1.4.1. Experimental session 1 (day 0)

Participants completed the SCL-90 and mood ratings at arrival in the laboratory. They then watched the trauma film, after which they completed mood ratings again. After that, intrusion diary A was given including standardized written information about how to record intrusions in the subsequent days until session 2. Thus, the last day of diary A ended when participants came to the lab for session 2. Participants had to read the instructions and asked for clarification if needed.

#### 1.4.2. Experimental session 2 (day 4)

Four days later participants returned to the laboratory for experimental session 2. They were assessed within a certain time window (e.g. not in the evening or early morning) to limit between-subject variation in day-length before and after the intervention. They were randomly allocated to one of the three experimental conditions just before their arrival. At arrival, participants handed in diary A and the experimenter checked all intrusions. Participants rated diary compliance after which the memory reactivation task was given. Participants in the reactivation+Tetris condition subsequently played Tetris for 10 minutes, those in the reactivation+Word games condition played the word game, and those in the reactivation-only control condition sat quietly for 10 minutes. During this 10-minute period, participants were asked to write down each intrusion using the pen and paper next to the computer. When the task had ended, participants rated task difficulty and pleasantness. Intrusion diary B was given (with the same instructions as for diary A) and participants were asked to record each intrusion in the subsequent days until session 3. Thus, the first day of diary B started immediately after the intervention.

#### 1.4.3. Experimental session 3 (day 7)

Participants handed in diary B and completed diary compliance. They were debriefed and received their reimbursement.

### 1.5. Analyses

Prior to analysing and unblinding the data, an analysis plan was formulated that specified what analyses would be carried out and which variable would be used as a primary outcome measure.

#### 1.5.1. Randomization check

A set of variables was selected on which the participants should not differ over the three experimental conditions: Age, Gender, SCL-90, Attention for the film, Attention for the memory reactivation stills, Distress prior to the film, Distress after the film, Compliance to diary A, and Compliance to diary B. Specifically, the pre to post increase in distress was calculated as a manipulation check because in all conditions the distress should increase after watching the film (Clark, Mackay, & Holmes, 2015). Bayes factors (BF0u) were calculated to test equality (H0) versus the unconstrained hypothesis (Hu).

#### 1.5.2. Hypotheses

Six informative hypotheses were formulated to address the three research questions according to a Bayesian approach, see Table 1.

Note that these hypotheses reflect the difference between diary B (first day) and diary A (last day), such that a < indicates relatively fewer intrusions. A comma indicates no constraints are posed between the adjacent parameters. For example, H1 should be read as: the reactivation+Tetris group has fewer intrusions in diary B relative to diary A compared to the reactivation-only control group, and no expectations are made regarding the changes in intrusion frequency in the Word game group. The hypotheses were evaluated in pairs, to answer the three research questions.

H1 vs. H6: Does reactivation+Tetris still lead to fewer intrusive memories than reactivation-only controls when applied four days after analogue trauma?

H2 vs. H5: Do reactivation+Tetris and reactivation+Word games lead to fewer intrusive memories than reactivation-only (intervention effect)?

H3 vs. H4: Does reactivation+Tetris lead to fewer intrusive memories than reactivation+Word games or vice versa (modality effect)?

**Table 1.** Informative hypotheses.^a^

H1: {µ_tetrisB_ – µ_tetrisA_ < µ_controlsB –_ µ_controlsA_}, µ_wordgamesB_ – µ_wordgamesA_^b^
H2: µ_tetrisB_ – µ_tetrisA_ = µ_wordgamesB_ – µ_wordgamesA_ < µ_controlsB –_ µ_controlsA_
H3: µ_tetrisB_ – µ_tetrisA_ < µ_wordgamesB_ – µ_wordgamesA_ < µ_controlsB –_ µ_controlsA_
H4: µ_wordgamesB_ – µ_wordgamesA_ < µ_tetrisB_ – µ_tetrisA_ < µ_controlsB –_ µ_controlsA_
H5: µ_tetrisB_ – µ_tetrisA_ = µ_wordgamesB_ – µ_wordgamesA_ = µ_controlsB –_ µ_controlsA_
H6: {µ_tetrisB_ – µ_tetrisA_ = µ_controlsB –_ µ_controlsA_}, µ_wordgamesB_ – µ_wordgamesA_

^a^All hypotheses reflect the difference between diary B (first day) and diary A (last day), so “<” indicates relatively fewer intrusions.

^b^A comma indicates no constraints are posed between the adjacent parameters.

#### 1.5.3. Bayes factors: hypothesis testing

For each research question the respective set of hypotheses was evaluated by means of a Bayes factor. A Bayes factor expresses the support for one hypothesis relative to another. For example, if BF12 is 1, both hypotheses are equally supported (i.e. no best hypothesis can be selected). If BF12 = 3, this means that H1 is three times more likely than H2 or three times less likely if BF12 = .33). Usually, a factor of 3 is interpreted as substantial evidence (Kass & Raftery, 1995), however, it is important to interpret the Bayes factor on a gradual scale. It expresses relative evidence, so a Bayes factor of 2.9 and 3.1 are in the same range of evidence, whereas a Bayes factor of 100 expresses really strong evidence.

#### 1.5.4. Bayes factors against Hu

After these planned comparisons, we investigated the relative evidence for all hypotheses. For this purpose, we calculated Bayes factors against the ‘baseline model’, i.e. the unconstrained hypothesis Hu (BFxu). The unconstrained hypothesis specifies no constraints on the parameters of interest. Note that these Bayes factors are obtained from the main analysis, but are only interpreted in second instance.

#### 1.5.5. Exploratory analyses: task difficulty and pleasantness

Because of the explorative character of the task difficulty and pleasantness analyses, relative evidence for all hypotheses was obtained by calculating Bayes factors for all possible hypotheses (Tetris is more/less/equally difficult/pleasant than Word Games) against the unconstrained hypothesis.

**BIEMS**. All analyses for the main and subsequent analyses were done in BIEMS (version 1.0.0.12; Mulder, Hoijtink, & de Leeuw, 2012). Any Bayesian analysis requires a prior distribution of the parameters. Specifying a prior distribution can be difficult, researchers want to refrain from adding subjective information in the analysis. BIEMS computes a prior distribution based on the data, resulting in a default Bayes factor. Part of the data is used to compute a conjugate prior and Bayes factors for all specified hypotheses against an unconstrained hypothesis.

## 2. Results

### 2.1. Randomization check

Means and standard deviations of all variables are listed in Table 2. The three groups seemed equal regarding age, gender, SCL-90, Distress before or after the film, Attention for the film, Attention for the memory reactivation stills, and diary B compliance (BF_0u_ between 1.85 and 5.96 for all variables). Groups seemed to differ on diary A compliance (BF0u = .07, i.e. difference was 14 times more likely than no difference): Compliance was lower in the reactivation+Tetris and reactivation-only control conditions than in the reactivation+Word games condition. Note that participants were not yet allocated to a condition at this point.

**Table 2.** Means (SDs) for the different conditions.

Variable	Reactivation+Tetris	Reactivation+Word games	Reactivation-only
**Randomization**			
Age	21.3 (2.8)	22.9 (3.1)	22.4 (5.0)
Gender *n* female (%)	9 (50%)	12 (67%)	14 (78%)
SCL90	118.5 (22)	126.0 (21.5)	121.1 (24.8)
Attention film	8.4 (1.2)	8.9 (.9)	8.4 (1.2)
Distress pre	1.4 (1.8)	1.4 (1.3)	2.0 (1.7)
Distress post	2.3 (2.4)	2.7 (2.4)	3.6 (2.3)
Compliance A	3.9 (3.1)	1.1 (1.4)	3.1 (2.5)
Intrusions diary A^a^	3.3 (3.1)	3.0 (3.0)	3.2 (3.0)
Attention reactivation^b^	9.1 (1.0)	9.3 (1.0)	9.2 (1.0)
Compliance B	1.7 (1.8)	2.2 (2.3)	2.3 (2.7)
**Intrusions**			
Intrusion-change^c^	.1 (.5)	.0 (.0)	.5 (.9)
Intrusions diary B^ad^	1.2 (1.7)	.3 (1.5)	1.1 (1.6)
**Explorative**			
Intrusions during task	1.9 (2.2)	1.9 (2.0)	4.3 (3.2)
Task difficulty	3.1 (2.3)	4.9 (2.1)	3.7 (2.9)
Task pleasantness	8.1 (1.7)	7.6 (1.4)	2.9 (2.1)

^a^Total number of intrusive memories reported in diary A or diary B.

^b^Attention to the memory reactivation stills.

^c^Change in intrusions from diary A to diary B (diary B/first day minus diary A/last day).

^d^The total number of intrusions in diary B is listed for clarity reasons and was not used as dependent variable for reasons explained in the text.

### 2.2. Intrusive memory changes from diary A to diary B

The results of the main analysis are depicted in Tables 3 and 4. Table 3 contains the Bayes factors for the three planned comparisons between the specific informative hypotheses to answer the three research questions (see Analyses). Table 4 contains the Bayes factors for all hypotheses against the unconstrained hypothesis (Hu).

**Table 3.** Bayes factors for the comparison of informative hypotheses in pairs regarding intrusion changes after the intervention (diaryB[1] – diaryA[4]).

Hypotheses	Bayes factors
BF16: H1 vs. H6^a^	6.05
BF25: H2 vs. H5	13.21
BF34: H3 vs. H4	.62 (1.61)^b^

^a^For example, BF15 expresses the support for H1 relative to H5.

^b^The entry within brackets is 1/BF, such that it expresses the support for H4 relative to H3.

**Table 4.** Bayes factors (BF) for all hypotheses against the unconstrained hypothesis (Hu).

Hypothesis	BFxu
H1	1.91
H2	2.47
H3	1.95
H4	3.16
H5	.19
H6	.31

#### 2.2.1. Effects for Tetris four days after analogue trauma

**Figure 1.**
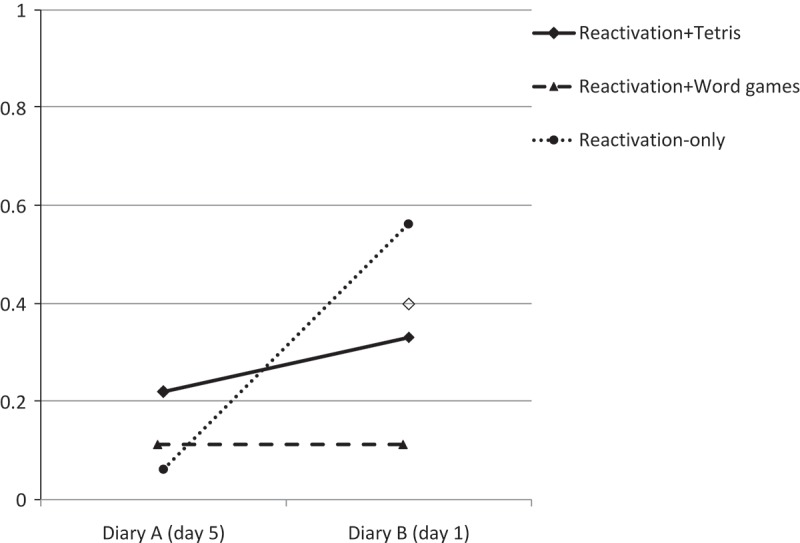
Intrusive memories of the film from before to after the intervention (diary A day 5 to diary B day 1).

For changes in intrusion frequency from diary A (last day) to diary B (first day) H1 was 6.05 times more supported than H6, indicating that reactivation+Tetris resulted in relatively fewer intrusive memories than reactivation-only (see Figure 1 for actual change scores in all groups).

#### 2.2.2. Intervention effects

H2 was 13.21 times more supported than H5, indicating an intervention effect: it is more likely that participants in the reactivation+Tetris and reactivation+Word games conditions had similar intrusion levels and intrusion frequencies in these two conditions were relatively lower than in the reactivation-only control condition, rather than all conditions leading to similar changes in intrusion frequency. Thus, an intervention seems more effective than no intervention.

#### 2.2.3. Modality effects

Finally, for the comparison of H3 and H4, H4 was slightly preferred (BF43 = 1.61). Thus, there is some evidence that reactivation+Word games resulted in relatively fewer intrusions than reactivation+Tetris. Note however, that a Bayes factor of 1.61 reflects weak evidence, and we cannot easily distinguish between H3 and H4.

#### 2.2.4. Bayes factors against Hu

Table 4 shows H4 and H2 received the strongest support against Hu, and are therefore the relative best hypotheses. Both were preferred over H3 and H1, which in turn were preferred over Hu. Note that H5 and H6 received less evidence than the baseline model, indicating that in these data, these hypotheses were highly unlikely. Thus, an intervention (reactivation+Tetris or reactivation+Word games) was more effective than reactivation-only: participants in the reactivation-only control condition have higher intrusion frequencies from diary A to diary B than participants in the two intervention conditions. Furthermore, H2 and H4 cannot be easily distinguished, indicating that either intrusion frequencies are similar for reactivation+Tetris and reactivation+Word games, or that reactivation+Word games leads to relatively fewer intrusions compared to reactivation+Tetris.

### 2.3. Exploratory analyses: task difficulty and pleasantness

Task difficulty (number of intrusions during the task and difficulty ratings) and pleasantness (pleasantness ratings) were explored as possible explanatory factors for intervention and modality effects. For all three variables, the same hypotheses are evaluated: Tetris ≤ Word games (Ha), Tetris ≥ Word games (Hb), Tetris = Word games (Hc). Thus, we tested whether Tetris was less difficult/pleasant than Word games (Ha), whether Tetris was more difficult/pleasant than Word games (Hb), or whether Tetris was equally difficult/pleasant as Word games (Hc).

#### 2.3.1. Task difficulty, intrusions during task

As can be seen in Table 5, the hypothesis that intrusion frequencies were equal during the two tasks received the highest support (BFcu = 1.74). BFs for Ha and Hb were comparable and received little support. Thus, it seems that intrusion frequencies during Tetris and Word games did not differ. However, the evidence is weak and no clear conclusion can be drawn for task Difficulty as indicated by intrusions during the task.

**Table 5.** Bayes factors (BF) for the exploratory analyses against the unconstrained hypothesis (Hu) for task difficulty and pleasantness.

Hypothesis	Difficulty/Intrusions^a^ BFxu	Difficulty/Ratings BFxu	Pleasantness/Ratings BFxu
Ha: µ_tetris_ < µ_wordgames_^b^	.96.	1.94	.42
Hb: µ_tetris_ > µ_wordgames_	1.04	.06	1.56
Hc: µ_tetris_ = µ_wordgames_	1.72	.32	1.20

^a^Number of intrusions during the task as an indicator of task difficulty

^b^< refers to Tetris being less difficult than Word games, (indicated by *more* intrusions during the task) and *lower* difficulty ratings.

#### 2.3.2. Task difficulty, ratings

As can be seen in Table 5, the hypothesis that Tetris was rated as less difficult than Word games (Ha) was most supported. The relative evidence was large; Hb and Hc did not receive much support. Thus, there is strong evidence for higher self-reported difficulty for Word games than Tetris.

#### 2.3.3. Task pleasantness, ratings

For task pleasantness, the hypothesis that is most supported is that Tetris is more pleasant than the Word games (Hb). Word games being more pleasant than Tetris (Ha) is unlikely. Both tasks being equally pleasant also receives some relative support, although less than Ha. Thus, it likely that Tetris was rated as more pleasant than Word games.

## 3. Discussion

The first aim of the present study was to examine whether previous effects of a visuospatial task (Tetris) after memory reactivation of analogue trauma could be replicated if the task was executed as long as four days after analogue trauma. Tetris indeed resulted relatively fewer intrusive memories than reactivation-only. That is, the participants that played Tetris after their memory of the film was reactivated four days after watching the film showed relatively fewer intrusive memories from the last day of diary A to the first day of diary B than participants who did not execute a task after their memory of the film was reactivated. Given the usual steep decline in intrusions in the days after a trauma film (e.g. James et al., 2015), the low intrusion frequencies at the time of our interventions and the restricted time period of the intrusion measure (last day of diary A to first day of diary B), it is notable that an effect was detected. Our finding is in line with previous findings and may indicate that visuospatial tasks may be quite strong in influencing memory (re)consolidation.

The second aim was to test an intervention effect. We found that intervening with either Tetris or Word games four days after the trauma film was effective: participants in both Tetris and Word games conditions had relatively fewer intrusions after the intervention than participants without a task. The evidence for this finding was strong. Note that the effects can be explained in two ways: executing a task after memory reactivation prevents an increase of intrusive memories, or memory-reactivation without a specific task results in an increase of intrusive memories. The results of the present study do not allow an answer to this question, as a fourth condition (no-reactivation/no-task) would be needed. Such a condition was included in a previous study that investigated intrusion development in the reconsolidation phase (James et al., 2015). It was found that participants in a no-reactivation/no-task group had similar intrusion frequencies in the days following the intervention as those in a reactivation/no-task condition, suggesting that no task with or without reactivation has the same effect. Our findings, as well as those of James et al. (2015) indicate that reactivation alone is insufficient to reduce intrusions. Note that the intervention in the study of James et al. (2015) was applied after 24 hours, when intrusion frequencies are still high, whereas we intervened after four days, when intrusion frequencies had approached 0 (for the course of intrusions after analogue trauma, see also James et al., 2015, experiment 1 and 2; Olatunji & Fan, 2015).

With respect to the third aim (modality effect), comparisons of the two task conditions showed a weak preference for Word games being more effective than Tetris. That is, having relatively fewer intrusions after playing Word games than after playing Tetris was 1.6 times more likely than the other way around (fewer intrusions after Tetris than after Word games). Posterior Model Probabilities also indicated that Word games being most effective of all conditions was the preferred hypothesis of all. However, note that the evidence was not strong. Thus, we found support for two hypotheses: visuospatial and verbal tasks were equally effective, or the verbal task was more effective than the visuospatial task. The interpretation of these results with respect to dual processing and working memory models is difficult. Support for the latter hypotheses (the verbal task being most effective) might confirm that task load is more relevant than task modality, especially since the verbal task was rated as more difficult than the visuospatial task, assumedly taxing the non-modal central executive more than the visuospatial task. However, task difficulty as reflected by intrusions during the tasks, was equal for both tasks. Also, self-rated task difficulty was clearly higher for the verbal tasks, but the effects on subsequent intrusion frequency were not clearly stronger. The former hypothesis (both tasks being equally effective) would support neither theoretical account. Original dual processing theories propose an enhanced effect for visuospatial tasks relative to verbal tasks, and working memory models propose an enhanced effect for the most difficult task. However, recent adaptations of PTSD models put less emphasis on the distinction between perceptual versus verbal modality but rather state that increased arousal and involvement facilitate perceptual processing, resulting in increased intrusion frequencies (Brewin & Burgess, 2014). In that light, both tasks could have worked as distractors, thereby interfering with perceptual processing, and leading to fewer intrusions. Note that the word games used here seem adequate in terms of effect on intrusions and are thought to tap into conceptual processing, though experimental tests are lacking in this respect. Naturalistic tasks as ours (rather than pure working memory or pure verbal tasks) may also draw on multiple modalities so it is unclear as yet precisely what balance of verbal, visuospatial and executive resources were being used to perform the task. Future studies may address the exact resources are taxed by these tasks. Also, being the first study using these word games, future research should elucidate whether Tetris and Word games are equally effective or Word games is the stronger intervention. A note of warning should be placed here as well, as some verbal tasks that have been used in the past *worsened* intrusion frequency (Bourne et al., 2010; Holmes et al., 2004), indicating one should be careful with implementing the use of verbal tasks in clinical practice.

Our study used a Bayesian approach for testing the hypotheses, which is different from previous frequentist analysis methods. A possible advantage of a Bayesian approach is that it provides *relative* evidence, i.e. relative strengths of an effect instead of finding total support or none at all. Such approach might also be meaningful with regard to testing competing meaningful hypotheses. For example, it is possible that two hypotheses are ‘very likely’ but one is ‘more likely’ than the other. In our case, we found most evidence for two hypotheses (*Tetris and Word games are both equally effective* and *Word games is the most effective task*), but could not distinguish between these two. Future research should further test these competing hypotheses against each other. A Bayesian approach might be useful in this light, as cumulative evidence allows incorporating previous findings in the statistical model (Konijn, Van de Schoot, Winter, & Ferguson, 2015).

Task difficulty was explored as an explanatory factor, because working memory theories would predict that resource competition is stronger for more demanding tasks, and thus effects would be larger. Thus, task difficulty is used as an index of working memory taxing. The results pointed at either both Tetris and Word games being equally difficult (intrusions during the task) or Word games (subjective ratings) being the most difficult. In a working memory framework, this would be in line with the finding that either Word games led to relatively fewer intrusions than Tetris, or both Tetris and Word games resulted in relatively fewer intrusions than reactivation-only controls. It is also in line with studies that found a dose–response relationship in that more taxing dual tasks resulted in greater declines in vividness and emotionality of voluntarily retrieved autobiographical memories (Engelhard, Van den Hout & Smeets, 201; Van den Hout et al., 2011), and fewer intrusive memories of aversive pictures (Pearson & Sawyer, 2011). Note that dual task effects on vividness and emotionality of autobiographical memories decreased with too much taxing, suggesting an inverted U-relationship (Engelhard et al., 2011; Van den Hout & Engelhard, 2012). Accordingly, non-movement (analogue to freezing, which is associated with a maximal attentional focus, thus optimal working memory capacity; Hagenaars, Oitzl, & Roelofs, 2014; Lang, Bradley, & Cuthbert, 1997) during a trauma film resulted in *increased* frequency of intrusive images of the film (Hagenaars et al., 2010, 2008). Note that there was no difference in the number of intrusions experienced during playing Tetris and Word games, yet participants rated Word games as more difficult than Tetris. Thus, either subjective difficulty ratings do not reflect actual difficulty levels, or intrusions during the task are not a good indicator for task difficulty (e.g. they could also be affected by task pleasantness), or difficulty is not a uniform concept.

Word games could be equally or slightly more effective because they are equally or more difficult than Tetris. Alternatively, Tetris and Word games could both be effective but for (partly) different reasons. Participants in our study enjoyed playing Tetris more than playing Word games. Possibly, this placed the analogue trauma memory in a different context once reactivated, after which the memory was reconsolidated with this new context. Similar to interventions such as imagery rescripting, counter-conditioning procedures and pre-extinction positive mood inductions, the pleasant context (Tetris) is proposed to be integrated in the new memory (Arntz, 2012; Engelhard, Leer, Lange, & Olatunji, 2014; Zbozinek et al., 2015).

Our study differed with previous studies on several points. Memory was reactivated with stills from the film that were characteristic for the scenes they were selected from. James et al. (2015) used images that depicted the moment just prior to the worst part of the scene. The latter method is also in line with human and animal conditioning studies, where a single unreinforced CS is usually used for memory reactivation (e.g. Nader, Schafe, & LeDoux, 2000; Schiller et al., 2010). The intervention is then placed during expectation of upcoming trauma, which fits with the view that ‘warning signals’ are a key component of a trauma memory. On the other hand, it has been argued that especially the most aversive scenes of a trauma should be activated in order to optimally change or contextualize the aversive memory. For example, Dibbets and Arntz (2016) found fewer intrusive memories for imagery rescripting after a trauma film if the most aversive moment of the scene was included in the script (versus a script that activated only the moments before the most aversive scene). Our scenes did not have a clearly preceding moment, as they depicted the aftermath of road traffic accidents and started after the trauma had taken place. Thus, there was no clear neutral ‘warning signal’ moment. In addition, reactivation took place long after the film was shown (four days later). Therefore, in this case, the most adequate way to reactivate the film-memory seemed to be activating a moment that was most typical for each scene. Relatedly, reactivation of the analogue trauma memory might have induced an increase of intrusive memories by ways of re-exposure (reinstatement). This would explain the increase in intrusions after the task in the reactivation-only control group, as well as the fast decline in the succeeding days (re-extinction). This might happen in real life too though, where trauma victims are often confronted with trauma reminders, which may trigger re-experiencing symptoms (APA, 2013; Ehlers & Clark, 2000; Foa, Steketee, & Rothbaum, 1989). At best, our findings might indicate that playing a game after memory reactivation hinders reinstatement (thus, the process of re-exposure triggering intrusive trauma memories).

Finally, note that in our study the task was executed after memory reactivation, without a time gap in between. Although a 10 minute time gap is sometimes used after memory reactivation (James et al., 2015; Schiller et al., 2010), effects have also been reported without a time gap (DeVietti, Conger, & Kirkpatrick, 1977) and there is no indication of exactly when the reconsolidation window might start. Limited evidence suggests that the end of the reconsolidation window is likely to be around six hours, but the neurobiology of reconsolidation is largely unknown and not allowing estimations about the start of such a window (e.g. Horne, Rodriguez, Wright, & Padilla, 1997; Rodriguez, Horne,  &  Padilla, 1999; Sara, 2000). In clinical practice and other experimental psychopathology, dual tasks interventions are usually administered during and immediately after memory reactivation (Van den Hout & Engelhard, 2012).

Our study has several limitations, such as the use of analogue trauma, limited sample size, and intervening when intrusion numbers are low (floor effects). Additionally, task difficulty was assessed indirectly. Future studies should use reaction time tasks to determine objective working memory load (Engelhard et al., 2011). Also, executing a task when intrusion frequencies are still relatively high (e.g. one of two days after analogue trauma) may allow actual intrusion declines from diary A to diary B. It may also allow including diary B in total. Finally, we found a difference in diary compliance. Note that this was in diary A, when participants were not yet allocated to a condition. Also, the reversed and counterintuitive wording of the answering scale might have elicited mistakes in scoring this question, which might be indicated by the fact that two participants scored extreme non-compliance on diary A only.

In sum, this study was the first to investigate the effects of two tasks applied four days after analogue trauma, after memory reactivation (i.e. proposedly having an impact during the reconsolidation window). The first hypothesis – that beneficial effects of Tetris would be found even four days after analogue trauma – was confirmed. We designed a new verbal task that had a game-aspect similar to Tetris (albeit on pen and paper), and was designed to be verbal and tap conceptual processing. Both the visuospatial and the new verbal task resulted in relatively fewer intrusions, even when applied four days after analogue trauma (intervention effect). Regarding modality effects, two hypotheses were supported: (1) the visuospatial task and the verbal task were equally effective and (2) the verbal task more effective. The verbal task was self-rated as more difficult and less pleasant than the visuospatial task, which may be in line with the working memory theory and resource competition. However, there were no differences in intrusions during the task. Further, naturalistic tasks as we used here are not pure working memory tasks, so we do not know for example how truly verbal the word games tasks was, as clearly it can also draw on visuospatial strategies – work calibrating the precise working memory demands of these tasks is needed before strong claims about modality could be made. The fact that the visuospatial task was rated as more pleasant may hint to another mechanism that merits further exploration. That is, other mechanisms may be at stake besides cognitive load and modality. The current findings merit replication as they might be relevant for real-trauma prevention interventions and extension to see if findings could be strengthened.
